# Global Warming and Dispersal Limitations Drive the Suitable Habitat Distribution of *Castanopsis indica*, *Castanopsis hystrix*, *Schima wallichii* Forest in China

**DOI:** 10.3390/plants15101432

**Published:** 2026-05-08

**Authors:** Huayong Zhang, Zhou Bian, Xiande Ji, Zhongyu Wang, Zhao Liu

**Affiliations:** 1 Research Center for Engineering Ecology and Nonlinear Science, North China Electric Power University, Beijing 102206, China; 120232232087@ncepu.edu.cn (Z.B.); zhy_wang@ncepu.edu.cn (Z.W.); 2 Theoretical Ecology and Engineering Ecology Research Group, School of Life Sciences, Shandong University, Qingdao 250100, China; liuzhao9555@sdu.edu.cn; 3 Energy Conversion Group, Energy and Sustainability Research Institute, Faculty of Science and Engineering, University of Groningen, Nijenborgh 6, 9747 AG Groningen, The Netherlands; x.ji.research@gmail.com

**Keywords:** global warming, dispersal limitations, *Castanopsis indica*, *Castanopsis hystrix*, *Schima wallichii* forest, optimised MaxEnt model, MigClim model

## Abstract

Global warming has increasingly threatened the suitable habitats of many species. However, ignoring dispersal limitations can substantially increase the uncertainty of species distribution predictions. This study employed optimised MaxEnt and MigClim models to explore the effects of global warming and dispersal limitations on the suitable habitat distribution of *Castanopsis indica*, *Castanopsis hystrix*, *Schima wallichii* (*C. indica*, *C. hystrix*, *S. wallichii*) forest in China. The results reveal that under current climatic conditions, this forest is mainly distributed in southwestern China. The key environmental factors influencing its distribution include isothermality, temperature seasonality, minimum temperature of the coldest month, and precipitation seasonality, among which temperature-related factors play a dominant role. Under future climate scenarios, the suitable habitat distribution of this forest is projected to expand overall and exhibit a northwestward migration trend. Notably, dispersal limitations significantly constrain the actual expansion of this forest, preventing it from keeping pace with climate change. The inclusion of dispersal limitations results in a contraction of the suitable habitat distribution of this forest under future climate scenarios, with the overall centroid migrating towards the southwest. In the future, *C. indica*, *C. hystrix*, *S. wallichii* forest will have some unoccupied suitable areas in China, which are primarily located north of its current suitable habitats. This study provides new insights for reducing uncertainties in species distribution predictions under climate change.

## 1. Introduction

The influence of global warming on species’ patterns of spatial distribution has become a focal point in ecology [1,2,3]. Rapid climate change modifies temperature and precipitation patterns, disrupting the climate suitability ranges to which species have long adapted and thereby driving species to respond to environmental change through the redistribution of their habitats [4,5,6]. Studies show that many species are migrating toward higher latitudes and elevations to track suitable climatic conditions [7,8,9]. As global warming continues to intensify, biodiversity is expected to decline further, placing some populations at risk of extinction and potentially impairing ecosystem functioning [6,10,11]. To address the challenges posed by global warming, predicting species’ suitable habitat distribution and migration trends under climate change has become an urgent task.

*Castanopsis indica*, *Castanopsis hystrix*, *Schima wallichii* (*C. indica*, *C. hystrix*, *S. wallichii*) forest represents one of the most characteristic vegetation types of subtropical evergreen broad-leaved forests. This type of forest is dominated by *C. indica*, *C. hystrix* and *S. wallichii* in its tree layer [12,13]. It is shade-tolerant, has a low tolerance to cold and drought, and favours warm and humid climates. In China, it is mainly distributed in regions west of the Ailao Mountains that are influenced by the southwest monsoon [14,15]. Due to its relatively rapid growth and strong soil and water conservation capacity, this forest can effectively control soil erosion and is therefore commonly used for timber forest and water conservation [15,16]. This vegetation type is essential to the regional cycling and storage of carbon as well as to maintaining forest ecosystem functions [17,18,19]. Compared with other forest types, it exhibits exceptionally high biodiversity and complex community structure, making it more sensitive to human activities and climate change [12,20,21]. At present, studies on *C. indica*, *C. hystrix*, *S. wallichii* forest mainly focus on individual species, particularly on species richness, genetic diversity, and population dynamics [13,22,23,24]. However, systematic analyses of the suitable habitat distribution of this forest under climate change remain limited, making it difficult to support the scientific conservation and sustainable utilisation of this vegetation resource.

Species distribution models have been widely employed to project the effects of future climate change on species ranges. These models simulate species distribution ranges through various algorithms by combining species occurrence records with essential climatic variables [20,25,26]. Among them, the MaxEnt model developed by Phillips shows notable advantages [27] and stands out among numerous modeling approaches. This model accurately estimates the probability of species occurrence under various environmental conditions by virtue of its high computational efficiency and flexibility, even when species records are incomplete and sample sizes are limited [27,28,29,30]. However, in most studies, the MaxEnt model assumes that species can disperse freely and primarily considers the effect of climatic factors on species distributions, while neglecting the effects of geographic barriers and species dispersal capacity [31,32,33]. As a result, the entire predicted suitable area is often regarded as the future distribution range of the species [34,35]. This assumption may lead to either overestimation or underestimation of future species distributions, thereby affecting the accuracy of predictions [36]. To correct this issue, Engler developed the MigClim model to simulate species dispersal patterns across different temporal scales [37]. The MigClim model is a spatially explicit dispersal model capable of simulating species migration under scenarios of climate change and landscape fragmentation [31,37]. The model is highly flexible and can be seamlessly integrated with the MaxEnt model. By combining potential habitat suitability maps with dispersal limitations, the MigClim model simulates the suitable habitats that species may actually occupy under climate change [38,39]. The coupled application of the MaxEnt and MigClim models provides an effective means for predicting species’ suitable habitats, fragmentation patterns, and migration dynamics.

This study employed vegetation distribution data alongside climatic, topographical and soil data. Utilising optimised MaxEnt and MigClim models, this study carried out systematic research into the suitable habitat distribution of *C. indica*, *C. hystrix*, *S. wallichii* forest in China. The study’s objectives are to: (1) assess the current suitable habitat distribution of *C. indica*, *C. hystrix*, *S. wallichii* forest and identify the main driving environmental factors influencing its distribution; (2) predict the suitable habitat distribution and migration trends of this forest under global warming; and (3) compare differences in the suitable distribution of this forest under climate change and dispersal limitations. The goal of this research is to offer a scientific foundation for the management and protection of *C. indica*, *C. hystrix*, *S. wallichii* forest in the face of global warming.

## 2. Results

### 2.1. Current Suitable Habitat Distribution and Identification of Environmental Factors

**Figure 1:**
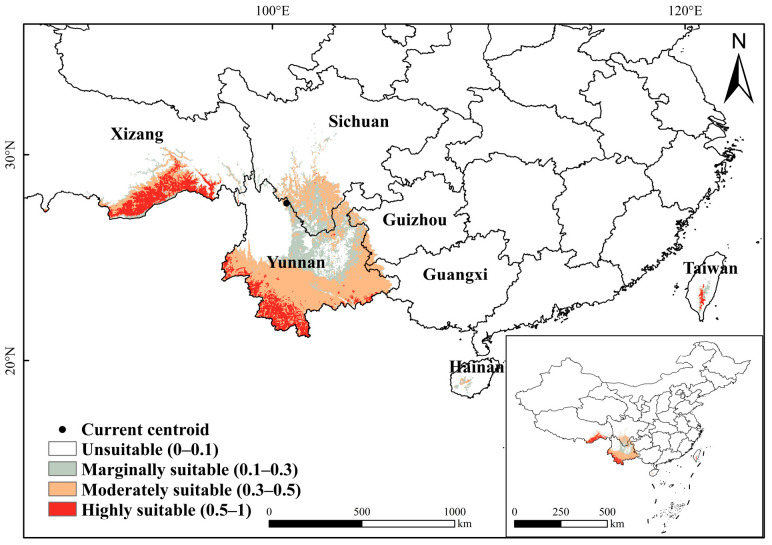
Suitable habitat distribution of *C. indica*, *C. hystrix*, *S. wallichii* forest under current climatic conditions. (Current centroid: Geometric centre of the current suitable habitat; the boundaries in the figure: Chinese provinces).

**Figure 2:**
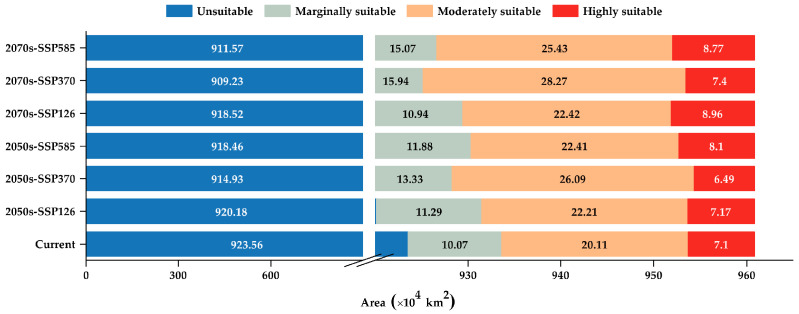
Suitable habitat area of *C. indica*, *C. hystrix*, *S. wallichii* forest under different climate scenarios. (The *x*-axis uses a break to accommodate the wide range of unsuitable habitat area).

The suitable habitat distribution of *C. indica*, *C. hystrix*, *S. wallichii* forest is mainly concentrated in southwestern China, with its centroid located in northern Yunnan Province (100.7° E, 25.49° N) (Figure 1). Under current climatic conditions, the total suitable habitat of *C. indica*, *C. hystrix*, *S. wallichii* forest accounts for approximately 3.88% of the total land area of China (Figure 2). The highly suitable habitat accounts for 19.04% of the total suitable habitat and is mainly concentrated in two regions. One region lies in southern Yunnan, in the Lancang River and Nujiang River areas west of the Ailao Mountains. The other region is situated in southern Xizang, in the Yarlung Zangbo River basin west of the Boshula Range and in the southeastern Himalayas. Scattered distributions are also found in Sichuan, Hainan, and Taiwan. The moderately suitable habitat covers a broader range, including Yunnan, Sichuan, Xizang, Guizhou, Guangxi, Hainan and Taiwan, accounting for 53.95% of the total suitable habitat. The marginally suitable habitat is primarily distributed across southern Sichuan and northeastern Yunnan, accounting for 27.01% of the total suitable habitat.

**Figure 3:**
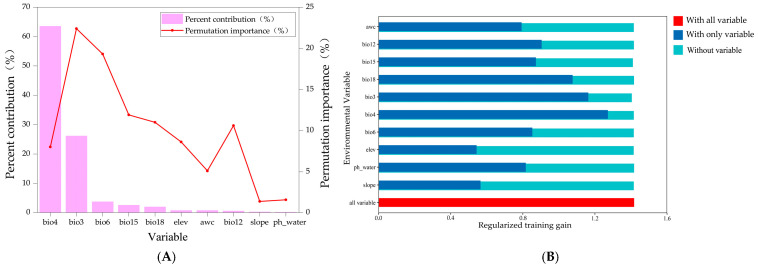
(**A**) The percentage contribution and permutation importance of environmental variables; (**B**) the results of the Jackknife test evaluating the environmental variables.

Among the ten environmental factors influencing the distribution of *C. indica*, *C. hystrix*, *S. wallichii* forest, climatic factors play a dominant role, whereas soil and topographic factors exert relatively minor effects on its distribution. Temperature seasonality (bio4) shows the highest contribution (63.5%), followed by isothermality (bio3, 26.1%), minimum temperature of the coldest month (bio6, 3.8%), and precipitation seasonality (bio15, 2.6%), with a cumulative contribution of 96%. In terms of permutation importance, the highest factor is isothermality (bio3, 22.4%), followed by minimum temperature of the coldest month (bio6, 19.3%) and precipitation seasonality (bio15, 11.9%). The cumulative permutation importance of these three environmental factors exceeds 50% (Figure 3A). When each environmental factor is used individually, temperature seasonality (bio4) produces the highest regularized training gain. It exerts the most pronounced influence on the distribution of *C. indica*, *C. hystrix*, *S. wallichii* forest (Figure 3B). Overall, isothermality (bio3), temperature seasonality (bio4), minimum temperature of the coldest month (bio6), and precipitation seasonality (bio15) are identified as the main environmental factors influencing the distribution of *C. indica*, *C. hystrix*, *S. wallichii* forest.

**Figure 4:**
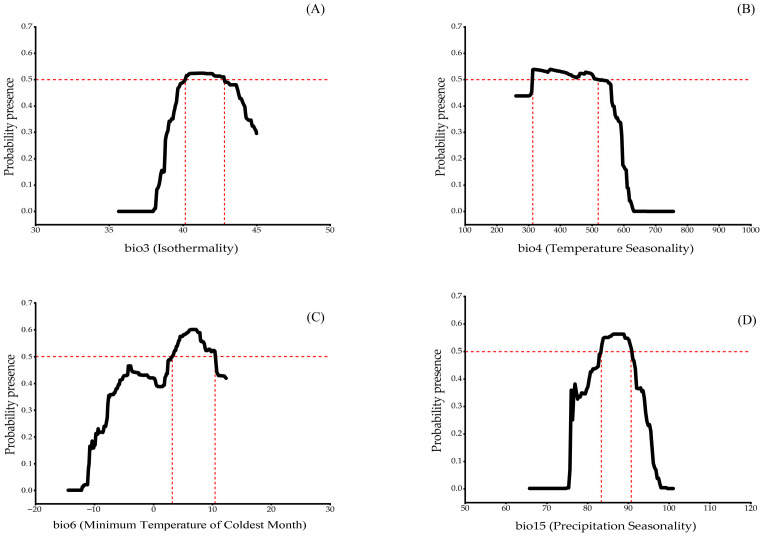
Response curves of the key environmental factors. (**A**) Isothermality; (**B**) Temperature seasonality; (**C**) Minimum temperature of the coldest month; (**D**) Precipitation seasonality.

The optimal ranges of the key environmental factors governing the distribution and growth of *C. indica*, *C. hystrix*, *S. wallichii* forest are as follows: isothermality (bio3), 40.15–42.80 (Figure 4A); temperature seasonality (bio4), 313.08–518.88 (Figure 4B); minimum temperature of the coldest month (bio6), 3.16–10.42 °C (Figure 4C); and precipitation seasonality (bio15), 83.36–90.70 (Figure 4D).

### 2.2. Shifts in Suitable Habitat Distribution and Centroid Migration

Under future climate conditions, the overall suitable habitat distribution of *C. indica*, *C. hystrix*, *S. wallichii* forest remains largely consistent with the current period. The range of suitable habitat for different suitability classes varies slightly under different climate scenarios (Figure S1).

**Figure 5:**
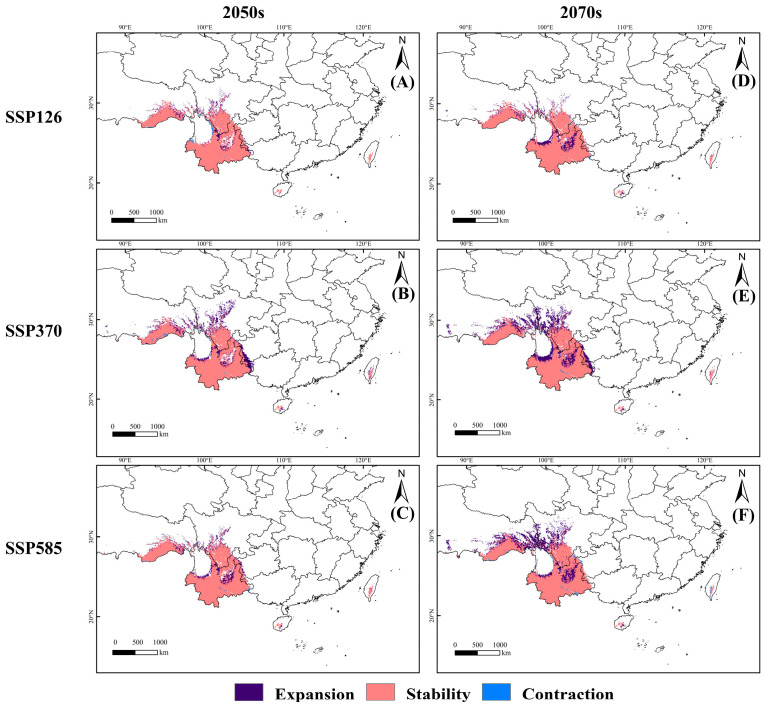
The expansion and contraction of the suitable habitat distribution of *C. indica*, *C. hystrix*, *S. wallichii* forest under different future climate scenarios. (**A**–**C**) 2050s projections under SSP126, SSP370, and SSP585 scenarios, respectively; (**D**–**F**) 2070s projections under SSP126, SSP370, and SSP585 scenarios, respectively.

**Figure 6:**
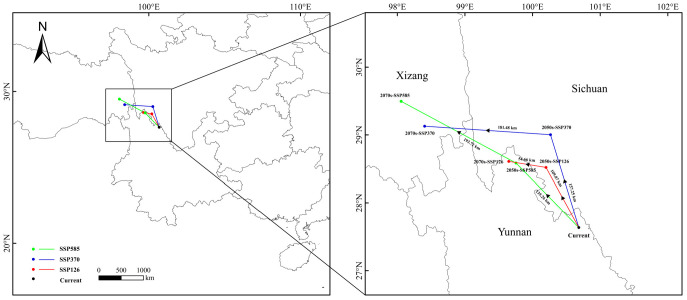
The migration routes of the suitable habitat centroid for *C. indica*, *C. hystrix*, *S. wallichii* forest in different climate scenarios.

Global warming drives the suitable habitat distribution of *C. indica*, *C. hystrix*, *S. wallichii* forest to exhibit an overall trend of expansion and migration toward northwestern higher-latitude regions (Table S1, Figure 5). The results show that under the SSP126 scenario, the suitable habitat area of *C. indica*, *C. hystrix*, *S. wallichii* forest increases over time. The suitable habitat area increases by 13.53% in the 2070s compared with the current period, with expansion mainly occurring in northern Yunnan. Centroid migration remains relatively moderate, shifting northwestward from Yunnan into Sichuan and subsequently moving further northwest back to northwestern Yunnan, with the migration distance gradually decreasing (Figure 6). Under the SSP370 scenario, the increase in the suitable habitat area of *C. indica*, *C. hystrix*, *S. wallichii* forest is substantially greater than that under the SSP126 and SSP585 scenarios. Over time, the suitable habitat area increases by 23.16% and 38.44% during the two future periods, respectively, with expansion mainly occurring in western Sichuan and across most regions of Yunnan. The centroid exhibits a continuous northward shift. From the current period to the 2050s, the centroid migrates northwestward to southwestern Sichuan, and by the 2070s it further shifts northwestward into Xizang. Compared with the SSP370 scenario, the suitable habitat area of *C. indica*, *C. hystrix*, *S. wallichii* forest decreases during both future periods under the SSP585 scenario. However, the northward expansion becomes more pronounced under this scenario, with the centroid continuously migrating toward higher latitudes, reaching the longest migration distance in the 2070s (Table S2). At the same time, northern Sichuan and Xizang emerge as the core zones of expansion.

### 2.3. Effects of Dispersal Limitations on Suitable Habitat Distribution

**Figure 7:**
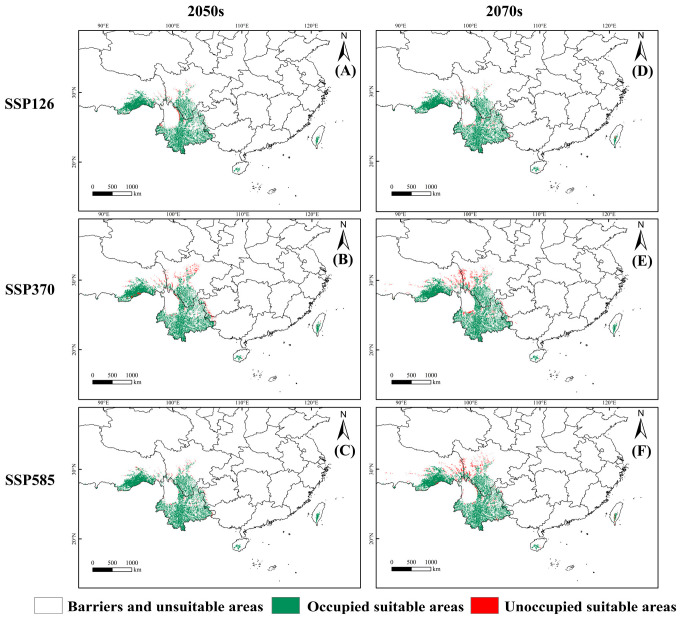
The occupied suitable areas of *C. indica*, *C. hystrix*, *S. wallichii* forest under future climate scenarios. (**A**–**C**) 2050s projections under SSP126, SSP370, and SSP585 scenarios, respectively; (**D**–**F**) 2070s projections under SSP126, SSP370, and SSP585 scenarios, respectively.

Dispersal limitations play an important role in shaping the actual distribution of *C. indica*, *C. hystrix*, *S. wallichii* forest. The suitable habitat area of this forest exhibits a decreasing trend under different future climate scenarios when dispersal limitations are incorporated (Figure 7). Under the SSP126 scenario, the suitable habitat area of *C. indica*, *C. hystrix*, *S. wallichii* forest decreases by 40.05% and 40.42% during the two future periods, respectively. Under the SSP370 scenario, the reduction in suitable habitat area is greater than that under the SSP126 and SSP585 scenarios. In particular, under the SSP370 scenario in the 2070s, the contraction is most pronounced, with a reduction rate reaching 46.64%. Under the SSP585 scenario, the suitable habitat of this forest contracts by 40.79–46.13% over time (Table S3).

**Figure 8:**
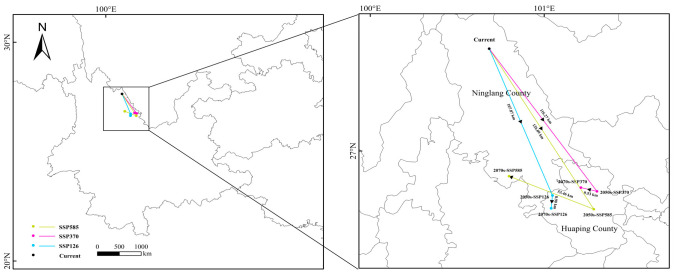
The migration routes of the suitable habitat centroid for *C. indica*, *C. hystrix*, *S. wallichii* forest under different climate scenarios after incorporating dispersal limitations.

Unlike the centroid migration projected for this forest under climate change alone, when dispersal limitations are considered, the suitable habitat centroid shifts generally toward the southwest, and future migration distances are gradually reduced (Figure 6 and Figure 8). However, the migration patterns differ among climate scenarios. Under the SSP126 scenario, the centroid shows an overall southeastward shift, eventually reaching Huaping County. Under the SSP370 scenario, the centroid migration trajectory first shifts southeastward and then deviates northwestward. Under the SSP585 scenario, the centroid initially moves southeastward and subsequently shifts back toward the northwest. Compared with the other two scenarios, the migration distance of the centroid is greatest under the SSP585 scenario (Table S4).

Although climate change can create new suitable habitats, dispersal limitations severely prevent *C. indica*, *C. hystrix*, *S. wallichii* forest from fully occupying these areas. This will cause the suitable habitat expansion and migration of this forest to lag far behind climate change. These unoccupied suitable areas are mainly located to the north of the current suitable habitat and account for 0.09–0.29% of the total land area of China (Table S3).

## 3. Discussion

The MaxEnt model has become a key tool for predicting species distribution [40,41]. Previous studies indicate that the MaxEnt model is typically implemented using default parameter settings, which may lead to excessive model complexity [42]. The regularization multiplier (RM) and feature combination (FC) are two key constraint parameters that control the level of model regularization [43]. Optimising these parameters can reduce model overfitting and sampling bias while improving the accuracy of species distribution predictions [44,45]. The optimisation results of this study show that when RM = 0.5 and FC = LQHPT, Δ*AICc* value is 0 (Figure S2A, Table S5), indicating that the model achieves the lowest level of overfitting under this parameter configuration. In this study, the AUC and TSS values were 0.889 (AUC > 0.8) and 0.759, respectively, demonstrating excellent predictive performance (Figure S2B, Table S6). Under current climatic conditions, the suitable habitat distribution of *C. indica*, *C. hystrix*, *S. wallichii* forest is mainly concentrated in southwestern China, including Yunnan, Xizang, and Sichuan. This prediction is largely consistent with the distribution range recorded in "Vegetation of China" [15], further confirming the high reliability of the model in predicting the distribution of this forest.

Climatic factors, particularly temperature and precipitation, are key drivers influencing plant physiological processes, distribution patterns, and phenological characteristics [46,47,48]. This study shows that temperature is the dominant factor (including bio3, bio4 and bio6) determining the distribution of *C. indica*, *C. hystrix*, *S. wallichii* forest. Of these, the high contribution of bio4 (63.5%) indicates that temperature seasonality is the primary driver influencing the distribution of this forest. This finding is consistent with results reported for other tree species in subtropical evergreen broad-leaved forests [49,50]. Temperature seasonality directly affects plant phenological patterns [51]. Excessive seasonal temperature fluctuations may lead to earlier flowering in some plant species, which can threaten their reproduction and growth and ultimately negatively affect population persistence [52]. Specifically, changes in the intensity of adverse seasons may further affect the degree of flowering synchrony and dispersion in Fagaceae plants (e.g., *C. indica* and *C. hystrix*) [53]. In addition, isothermality (bio3) also plays an important role in shaping the distribution of this forest. As the factor with the highest permutation importance (22.4%), bio3 reflects the ratio between the diurnal temperature range and the annual temperature range. According to the response curve analysis, the optimal range of isothermality for this forest is 40.1–42.8. This range closely corresponds to the monsoon climate characteristics of southwestern China, where winters are mild, summers are relatively cool, and daily temperature fluctuations remain relatively stable. Such stable microclimatic conditions favour evergreen broad-leaved tree species by supporting continuous photosynthesis and carbon accumulation throughout the year [54,55].

Precipitation also affects the distribution of *C. indica*, *C. hystrix*, *S. wallichii* forest to a certain extent. In this study, precipitation seasonality (bio15) still shows relatively high permutation importance (11.9%). This finding is consistent with the results reported by Dong [56]. The seasonal distribution of precipitation is a key factor determining vegetation suitability [57,58]. When precipitation decreases markedly during the dry season, soil moisture stress can inhibit nutrient uptake by roots, thereby affecting carbon allocation in trees [59]. This effect is particularly pronounced for evergreen broad-leaved tree species that depend on a stable water supply. Increased precipitation seasonality may disrupt their physiological balance and limit their expansion into seasonally dry regions [49,60]. The combined importance of temperature and precipitation reflects the integrated hydrothermal requirements of *C. indica*, *C. hystrix*, *S. wallichii* forest. Its growth cycle requires both sufficient water availability and suitable thermal conditions. Therefore, the growth and distribution of *C. indica*, *C. hystrix*, *S. wallichii* forest result from the combined effects of multiple climatic factors, rather than being driven by a single environmental factor.

Numerous studies show that global warming may drive some species to expand and migrate toward northern or higher-latitude regions [7,61,62]. This pattern is highly consistent with our findings. Under climate warming, the suitable habitat distribution of *C. indica*, *C. hystrix*, *S. wallichii* forest is generally projected to expand toward the northwest. This phenomenon may be attributed to the increase in minimum temperature caused by climate warming, which alleviates the low-temperature stress experienced by subtropical evergreen broad-leaved tree species and enables them to surpass the threshold imposed by extreme winter cold on their geographic distribution [63,64]. Notably, the conclusions of this study differ from those reported in a study on *Ziziphus jujuba* var. *spinosa*. Zhao proposed that the degree of habitat expansion of this species increases continuously with rising carbon emission concentrations [65]. By comparison, the magnitude of suitable habitat expansion for *C. indica*, *C. hystrix*, *S. wallichii* forest in this study was lower under the high-emission SSP585 scenario relative to the moderate SSP370 scenario, showing a clear declining trend in expansion magnitude under intensified climate change. Heat stress can reduce carbon use efficiency during photosynthesis, thereby lowering growth rate, which in turn restricts habitat expansion and may even lead to the loss of some habitats [66,67]. Accordingly, we speculate that the temperature rise caused by increasing carbon emissions may have approached or even exceeded the heat tolerance limit of this forest, potentially leading to a reduction in its suitable habitat. Furthermore, under high-emission scenarios, the frequency of extreme weather events, particularly droughts, is predicted to rise. This may lead to imbalances in precipitation seasonality, ultimately affecting the stability of suitable habitats.

Previous studies predicting species distributions have regarded climatic factors as the core drivers of changes in their distribution ranges [68,69]. However, some scholars have pointed out that dispersal limitation also plays an important role in shaping species distribution ranges, which may result in the realised niche being smaller than the fundamental niche [70,71]. The results of this study show that under different future climate scenarios, the actual suitable habitat distribution area of *C. indica*, *C. hystrix*, *S. wallichii* forest is significantly smaller than its potential suitable habitat distribution, with 0.09–0.29% of suitable areas remaining unoccupied. This finding indicates that although climate warming creates new adaptive areas, dispersal limitations prevent this forest from fully occupying these regions within the simulated time frame. This result is consistent with previous studies [20,72]. Unlike the northwestward centroid migration predicted under climate change alone, the inclusion of dispersal limitations shows that the centroid of the suitable habitat of *C. indica*, *C. hystrix*, *S. wallichii* forest shifts overall toward the southwest. Moreover, under different future climate scenarios, the migration distance of the centroid is shorter than that predicted under climate change alone. Several studies show that the seeds of *C. indica*, *C. hystrix*, *S. wallichii* forest are mainly dispersed by gravity, resulting in limited dispersal distances that generally do not exceed the canopy radius of the parent tree [73,74,75,76]. We surmise that this short-distance dispersal pattern severely constrains the rapid expansion of this forest into newly adaptive areas, preventing it from keeping pace with climate change and ultimately leading to migration lag. In recent years, with the transformation of land use patterns, particularly the high-intensity interventions resulting from the conversion of forests to other land types by humans, its natural habitats are being destroyed and progressively fragmented [20]. This shift in patterns creates physical barriers that hinder seed dispersal, thereby altering species’ geographic distribution patterns and reducing community regeneration and resistance to disturbance [77,78]. Therefore, the implementation of assisted migration strategies, such as the construction of ecological corridors, can effectively connect isolated habitat patches, facilitate species dispersal and gene flow, and significantly reduce the extinction risk of this forest under climate change [79,80,81]. Based on actual records of seed dispersal, we have assumed that the model simulates dispersal only over short distances. However, there are always occasional dispersal events in nature, which may introduce a degree of uncertainty. In the future, long-term field monitoring should be carried out to record these sporadic events, and consideration should be given to incorporating them into model applications. Such efforts would enable more accurate predictions of the actual dispersal potential and adaptive capacity of *C. indica*, *C. hystrix*, *S. wallichii* forest, thereby providing a scientific basis for its conservation and adaptive management.

In this study, our species data were obtained from the officially standardised Vegetation Atlas of China, reliably representing the geographical distribution of *C. indica*, *C. hystrix*, *S. wallichii* forest. However, this dataset may introduce positional uncertainty and potential pseudo-replication. Future research could conduct additional field surveys and adopt ground-verified occurrence records to improve data accuracy in subsequent distribution modelling [82]. In addition, this study only used a single model (BCC-CSM2-MR). Given that projections of future hydrothermal patterns and magnitudes of change vary among global climate models (GCMs) due to their inherent differences, this introduces uncertainty into species distribution predictions [83]. Subsequent studies could integrate ensemble projections from multiple climate models, thereby further reducing the uncertainty associated with climate models.

## 4. Materials and Methods

### 4.1. Data Acquisition and Processing

**Figure 9:**
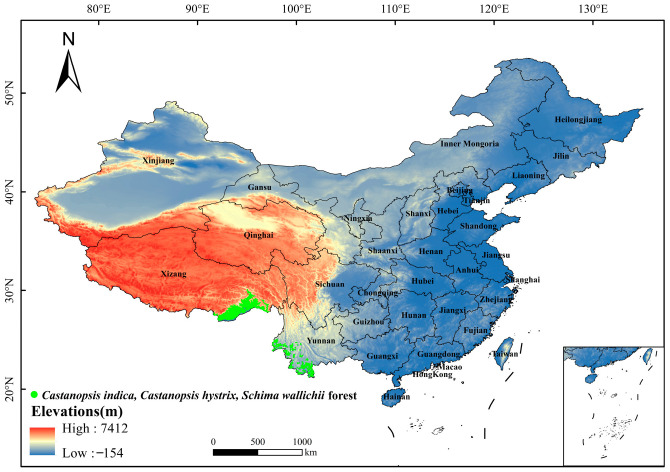
Distribution of sample points of *Castanopsis indica*, *Castanopsis hystrix*, *Schima wallichii* forest in China.

In this study, the base map boundary data for China were sourced from the National Geomatics Center of China, with the map approval number GS (2024) 0650 [84]. The distribution data of *C. indica*, *C. hystrix*, *S. wallichii* forest were derived from the “Vegetation Atlas of China (1:1,000,000)” published by Science Press in 2001 [85]. Using ArcGIS 10.8.2, patch data of this forest were extracted from the above dataset and converted into raster data. The Raster to Point tool was then applied to transform raster data into point data with a spatial resolution of 1 km × 1 km. To avoid spatial autocorrelation caused by overly close sample points, ENMTools (version 1.3) was used to remove redundant occurrence records, ensuring that only one occurrence point was retained within each 2.5′ × 2.5′ grid. After filtering, 3663 sample points were retained (Figure 9).

Climate and elevation data were obtained from WorldClim version 2.1 with a spatial resolution of 2.5′ (https://worldclim.org (accessed on 27 December 2024)). The climate dataset includes the current period (1970–2000) and two future periods: the 2050s (2041–2060) and 2070s (2061–2080), with 19 bioclimatic variables available each period. Slope and aspect data were derived from the elevation dataset using the “Surface Analysis tool” in ArcGIS 10.8.2. Soil data included six soil variables and were derived from the Harmonized World Soil Database (HWSD 2.0), jointly developed by the Food and Agriculture Organization of the United Nations (FAO) and the International Institute for Applied Systems Analysis (IIASA), with a spatial resolution of 1 km (https://gaez.fao.org/pages/hwsd (accessed on 27 December 2024)). The land-use data used in the MigClim model were obtained from China’s Multi-Period Land Use Land Cover Remote Sensing Monitoring Dataset (CNLUCC) provided by the Resource and Environmental Science Data Center of the Chinese Academy of Sciences, with a spatial resolution of 30 m (https://www.resdc.cn (accessed on 27 December 2024)).

The BCC-CSM2-MR global circulation model, developed by the China Meteorological Administration (CMA), performs well in simulating the climate of China and provides relatively reliable climate projections [86]. For future climatic conditions (2050s and 2070s), this study selected three Shared Socioeconomic Pathways (SSPs) for simulation: SSP126 (low-emission scenario), SSP370 (medium-emission scenario), and SSP585 (high-emission scenario). Due to the lack of data on future topography and soils under climate change, this study assumed these two variables remained constant throughout the simulation.

Using ArcGIS 10.8.2, the environmental variables used in the MaxEnt and MigClim models were uniformly preprocessed, and the spatial resolution was ultimately standardized to 2.5′. To reduce model overfitting caused by multicollinearity among environmental variables, we screened the 28 initially selected environmental variables (Table S7). The screening process is as follows: (1) we incorporated the 28 environmental variables and species data into the MaxEnt model (version 3.4.4) for operation, and removed environmental variables with a contribution rate of 0% according to the output results. (2) We conducted Pearson correlation analysis for environmental variables using ENMTools (version 1.3) (https://www.enmtools.com/ (accessed on 28 December 2024)) (Figure S3). When two variables showed strong correlation (|r| > 0.8), the variable with the lower contribution was removed. This correlation threshold (|r| > 0.8) has been widely adopted in numerous species distribution modelling studies to mitigate multicollinearity [87,88]. After this screening process, 10 environmental variables were retained for the final model analysis (Table 1).

### 4.2. Calculation of Suitable Habitat Distribution and Centroid Migration

In this study, the ENMeval 2.0 package in R version 4.3.3 (https://github.com/jamiemkass/ENMeval (accessed on 28 December 2024)) was used to optimise the RM and FC of the MaxEnt model to determine the optimal model configuration [89,90]. First, the distribution data of *C. indica*, *C. hystrix*, *S. wallichii* forest were randomly divided into a training dataset (75%) and a testing dataset (25%). RM values were varied from 0.5 to 4.0 in increments of 0.5, and five feature combinations were evaluated: linear (L), linear–quadratic (LQ), linear–quadratic–hinge (LQH), linear–quadratic–hinge–product (LQHP), and linear–quadratic–hinge–product–threshold (LQHPT). Finally, the corrected Akaike Information Criterion (AICc) (a revised version of Akaike’s Information Criterion for small sample sizes) was used to evaluate the model fit. The parameter combination with the lowest AICc value was selected as the optimal configuration for the model (Δ*AICc* = 0) [91].

The processed occurrence data of *C. indica*, *C. hystrix*, *S. wallichii* forest and the environmental variable data were imported into the optimised MaxEnt model for modelling. The maximum number of iterations and background points were set to 500 and 10,000, respectively. Meanwhile, 75% of the occurrence records were randomly selected as the training dataset, and the remaining 25% were used as the testing dataset. The model outputs were generated using 10 replicates with cross-validation, and the output format was set to Logistic. The performance of the MaxEnt model was evaluated using the area under the receiver operating characteristic curve (AUC). To further enhance the model’s prediction accuracy, the true skill statistic (TSS) was calculated. TSS is a metric used to assess the performance of classification models. According to the study by Allouche, the True Skill Statistic (TSS) ranges from −1 to +1, and values closer to +1 represent better predictive performance [92]. The AUC value typically ranges from 0 to 1. In general, AUC < 0.5 indicates poor predictive performance, whereas AUC > 0.8 indicates good model performance [93,94].

The Reclassify tool in ArcGIS was used to categorise the suitable habitat distribution of *C. indica*, *C. hystrix*, *S. wallichii* forest. Based on the threshold selection adopted for other similar tree species in subtropical evergreen broad-leaved forests [61,95], the suitable habitats were divided into four classes: unsuitable (0–0.1), marginally suitable (0.1–0.3), moderately suitable (0.3–0.5), and highly suitable (0.5–1) [96]. The “Distribution Changes Between Binary SDMs” in the SDM Toolbox was then applied to compare and quantify the changes in the area and spatial extent of the suitable habitat distribution of *C. indica*, *C. hystrix*, *S. wallichii* forest under current and future climate scenarios, thereby identifying the expansion and contraction of its suitable habitat [97]. Meanwhile, the “Centroid Changes (Lines)” tool in the same toolbox was used to calculate the centroid location of the suitable habitat distribution and to analyse its migration trends.

### 4.3. Identification and Calculation of Driving Environmental Factors

In this study, we comprehensively evaluated the percentage contribution, permutation importance, and jackknife test results of environmental variables to identify the main environmental factors influencing the distribution of *C. indica*, *C. hystrix*, *S. wallichii* forest. Using 0.5 as the threshold to determine species presence versus absence has been widely applied in ecology [98]. As recommended by Phillips’ research, environmental variable values corresponding to an occurrence probability of *p* ≥ 0.5 are considered indicative of favourable conditions for species growth [30]. To further elucidate the influence of environmental factors on the distribution of this forest, an occurrence probability threshold of *p* ≥ 0.5 was applied to delineate favorable response intervals. Response curves for the primary driving factors were subsequently generated to characterise the optimal ranges of environmental variables associated with suitable growth conditions for this forest.

### 4.4. Calculation of Dispersal Limitations

In this study, the MigClim package in R version 4.3.3 (https://github.com/robinengler/MigClim (accessed on 28 December 2024)) was used to simulate the suitable habitat distribution of *C. indica*, *C. hystrix*, *S. wallichii* forest under dispersal limitations by integrating land-use data with habitat suitability maps generated by the MaxEnt model. The MigClim model incorporates the combined effects of climate change, species dispersal, and geographic barriers, enabling a more accurate representation of future spatial distribution patterns [31,39]. In the MigClim model, species dispersal is represented by two types: short-distance dispersal (SDD) and long-distance dispersal (LDD). The former describes a process in which most seeds are dispersed according to a relatively predictable pattern, whereas the latter affects only a small proportion of seeds and follows a more random dispersal pattern. Seeds of *C. indica* and *C. hystrix* fall directly from parent trees, with gravity as the primary dispersal mode [73,99,100]. Secondary dispersal is mainly conducted by small rodents, transporting seeds only over short distances (e.g., the mean dispersal distance of *C. indica* seeds by rodents is 6.8 m, mostly within 20 m) [101]. Transported seeds are eventually fed on by rodents, resulting in low seedling survival rates. *S. wallichii* relies on wind dispersal. Setiawan’s study indicated that only a very small number of seeds disperse beyond 5 m from the afforestation area [102]. Therefore, long-distance dispersal was not considered in this study, and the simulations were conducted only under short-distance dispersal.

Following the approaches of Zhao and Li [57,103], several datasets were required as inputs for the MigClim model. The current habitat suitability map generated by the MaxEnt model was used as the initial distribution (iniDist). Habitat suitability maps representing the period from the current climate to the 2050s or 2070s were used as hsMap1 and hsMap2, respectively. Extensive anthropogenic land use reduces the functional connectivity between forest patches and acts as a natural barrier to tree seed dispersal [104]. In this study, we defined farmland and urban land extracted from land use data as seed dispersal barriers, and performed binary processing on the obtained rasters. In addition, a series of dispersal parameters were specified in the model (Table 2). Finally, according to the status of grid cells at the end of the simulation, the raster outputs generated by the MigClim model were reclassified into three categories: barrier and unsuitable areas, occupied suitable areas, and unoccupied suitable areas.

## 5. Conclusions

This study integrated the optimised MaxEnt model and MigClim model to assess, under the BCC-CSM2-MR model, the impacts of climate change on the suitable habitat distribution of *C. indica*, *C. hystrix*, *S. wallichii* forest under current and future climate scenarios, and to compare the differences in distribution patterns driven by warming and dispersal limitations. The main conclusions are as follows: (1) Under current climatic conditions, *C. indica*, *C. hystrix*, *S. wallichii* forest is mainly distributed in southwestern China, with core areas located in southern Yunnan and southeastern Xizang. Temperature emerges as the primary climatic driver governing the suitable habitat distribution of this forest. In particular, isothermality and temperature seasonality are the key factors influencing the current and future suitable habitat distribution of this forest, whereas precipitation plays a secondary regulatory role. (2) Under the effect of global warming, the suitable habitat area of this forest exhibits an overall expansion trend, and the centroid of its suitable habitat tends to migrate toward northwestern higher latitudes. Compared with the SSP126 and SSP585 scenarios, the SSP370 scenario is more favorable for the expansion of the suitable habitat of this forest. Under the SSP370 scenario, its suitable habitat area increased by 23.16% in the 2050s and by 38.44% in the 2070s. (3) Dispersal limitations substantially constrain the actual expansion of *C. indica*, *C. hystrix*, *S. wallichii* forest, making its migration rate unable to match the pace of climate change and thereby resulting in migration lag. After incorporating dispersal limitations, the suitable habitat area of this forest decreases under different future climate scenarios, and the centroid of its suitable habitat shifts overall toward the southwest, with a significantly shorter migration distance. Furthermore, under all future climate scenarios, 0.09–0.29% of suitable areas for this forest in China will remain unoccupied. This study emphasises that both climate change and dispersal limitations should be jointly considered in biodiversity conservation planning. It not only provides new insights into the response mechanisms of *C. indica*, *C. hystrix*, *S. wallichii* forest to environmental change, but also offers scientific support for the development of conservation and management strategies for forest vegetation in the context of global warming.

## Figures and Tables

**Table 1:** Environmental variables for the MaxEnt model construction.

Symbol	Variables Name	Unit
bio3	Isothermality	unitless
bio4	Temperature seasonality	unitless
bio6	Minimum temperature of the coldest month	°C
bio12	Annual precipitation	mm
bio15	Precipitation seasonality	unitless
bio18	Precipitation of the warmest quarter	mm
elev	Elevation	m
slope	Slope	°
awc	AWC for rootable soil depth	mm
ph_water	pH in water	−log(H+)

**Table 2:** Parameter settings and interpretations in the application of the MigClim model.

Parameters	Explanation	Parameter Settings
rcThreshold	Habitat suitability threshold	100
dispSteps	The number of dispersal steps to simulate within each environmental change step	25/35
envChgSteps	The number of environmental change steps to simulate	2
dispKernel	Probability of an occupied cell dispersing propagules as a function of distance	1
iniMatAge	The initial maturity age of newly colonized cells	1
propaguleProd	The probability function of cells producing propagules over time	c (0.01, 0.08, 0.5, 0.92)
lddFreq	The probability for an occupied cell to produce a long-distance dispersal event	0
replicateNb	Number of model simulations	10

## Data Availability

All links to input data are reported in the manuscript and all output data are available upon request to the authors.

## Supplementary Materials

The following supporting information can be downloaded at: https://www.mdpi.com/article/10.3390/plants15101432/s1, Figure S1: Suitable habitat distribution of *C. indica*, *C. hystrix*, *S. wallichii* forest under future climatic conditions; Figure S2: (A) Results of optimizing MaxEnt model parameters by the ENMeval package; (B) MaxEnt model’s receiver operating characteristic curve (ROC); Figure S3: Pearson correlation heatmap of 28 environmental variables; Table S1: The area and changes in the suitable habitat distribution of *C. indica*, *C. hystrix*, *S. wallichii* forest under different climate scenarios; Table S2: Centroid coordinates and migration distance of *C. indica*, *C. hystrix*, *S. wallichii* forest under climate change; Table S3: The suitable habitat distribution of *C. indica*, *C. hystrix*, *S. wallichii* forest after incorporating dispersal limitations; Table S4: Centroid coordinates and migration distance of *C. indica*, *C. hystrix*, *S. wallichii* forest with dispersal limitations incorporated; Table S5: Comparison of the results of the MaxEnt model parameter optimization; Table S6: AUC and TSS values of the optimised MaxEnt model; Table S7: 28 initial environmental variables in the application of the MaxEnt model.
