# Supplementary material for: Global Warming and Dispersal Limitations Drive the Suitable Habitat Distribution of Castanopsis indica, Castanopsis hystrix, Schima wallichii Forest in China
Source: Plants (Basel). 2026 May 8;15(10):1432. doi: 10.3390/plants15101432 (PMC13210694; doi:10.3390/plants15101432)
Supplement: Supplementary file 1 [file plants-15-01432-s001.zip › plants-4260709-supplementary.pdf]

Supplementary Materials

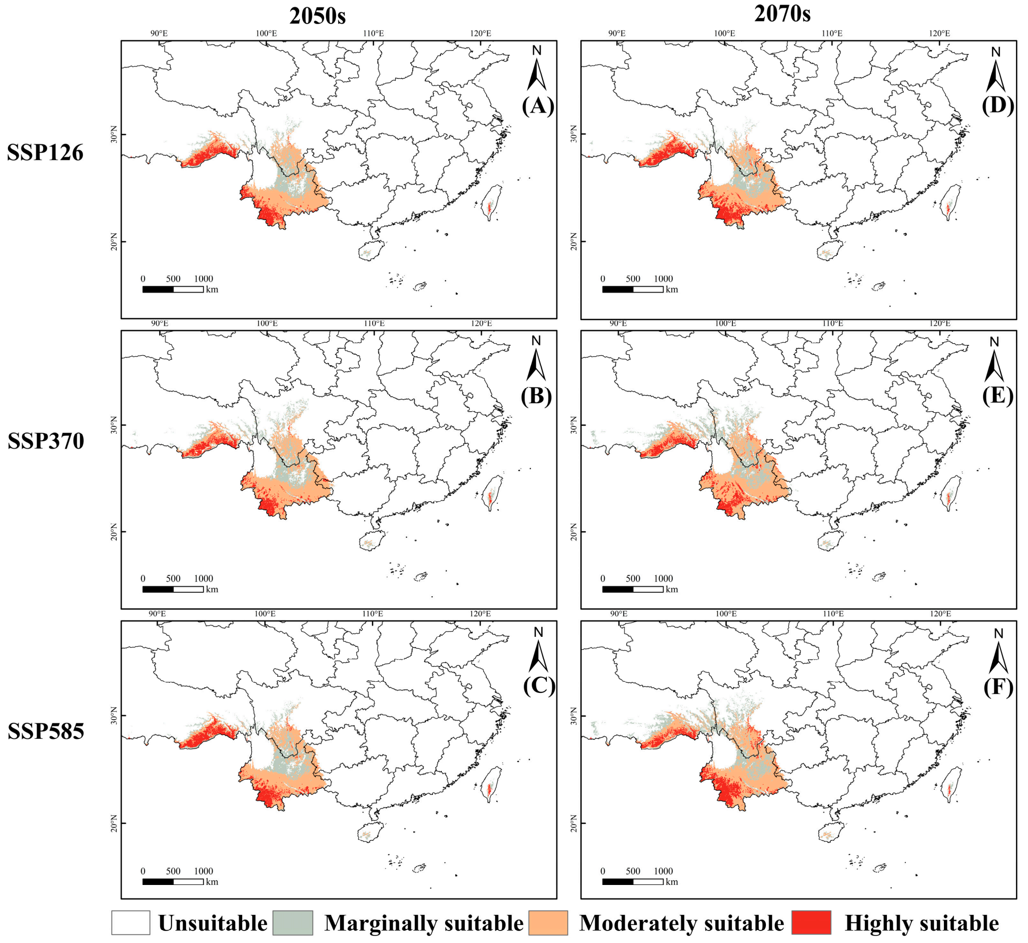

Figure S1. Suitable habitat distribution of *C. indica*, *C. hystrix*, *S. wallichii* forest under future climatic conditions.

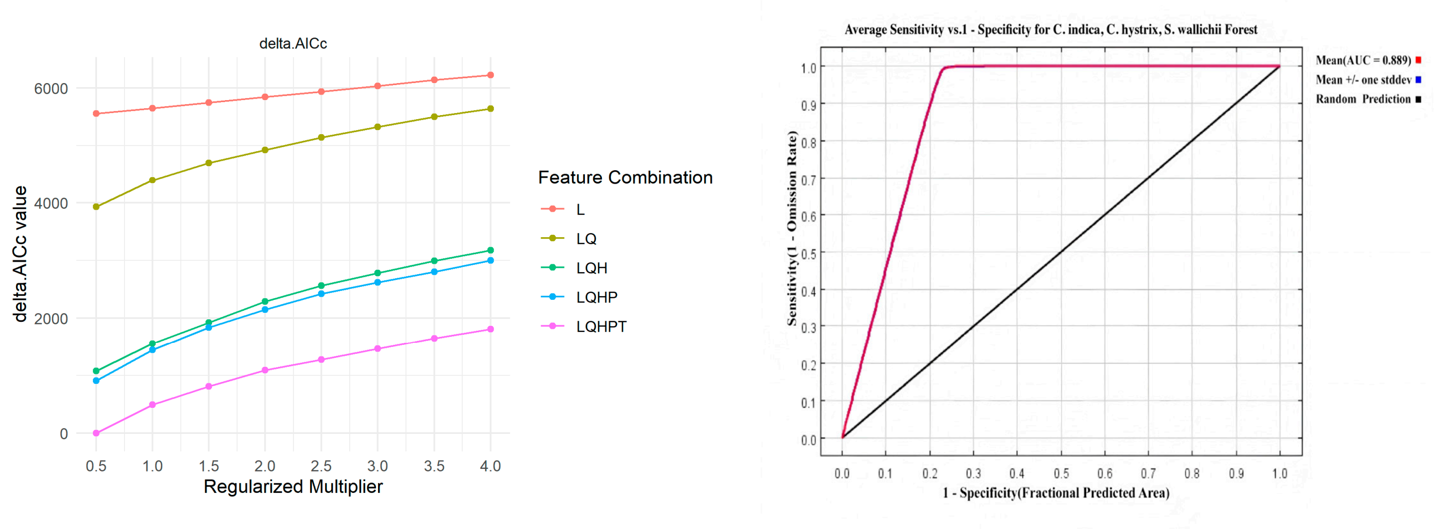

(A) (B)

Figure S2. (A) Results of optimizing MaxEnt model parameters by the ENMeval package; (B) MaxEnt model's receiver operating characteristic curve (ROC).

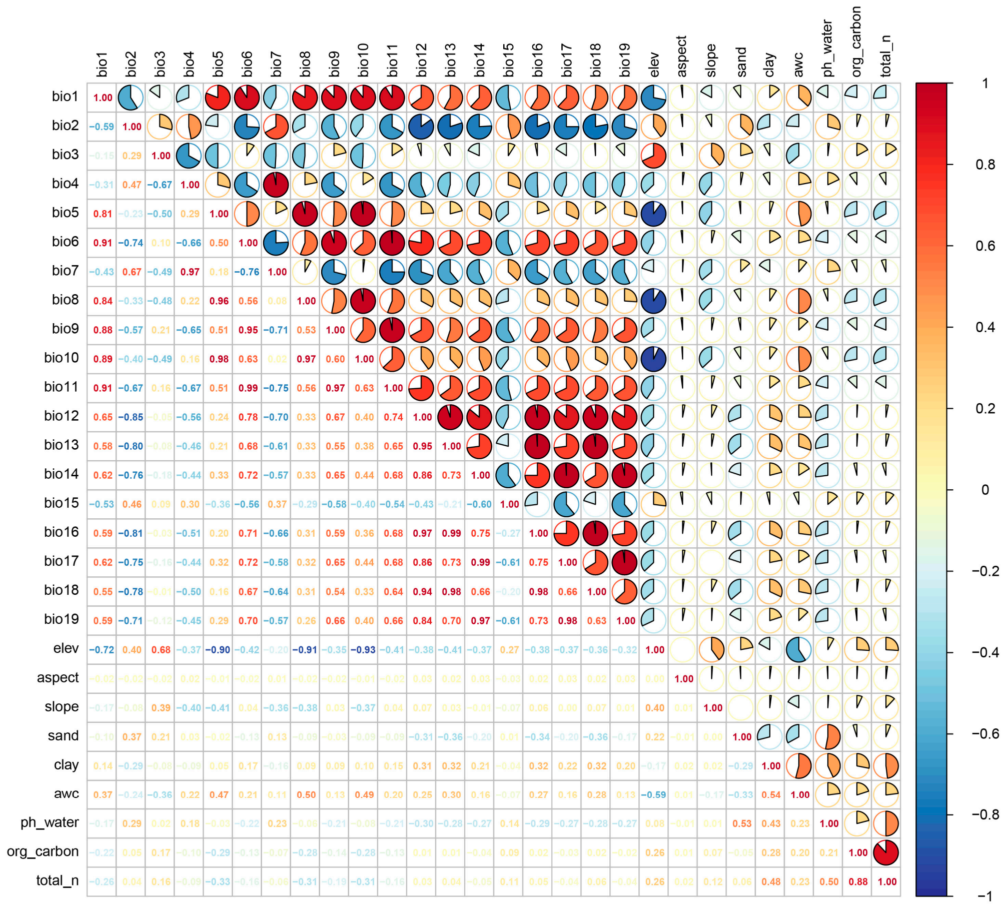

Figure S3. Pearson correlation heatmap of 28 environmental variables.

Table S1. The area and changes in the suitable habitat distribution of *C. indica*, *C. hystrix*, *S. wallichii* forest under different climate scenarios.

Scenarios	Periods	Marginally suitable habitat		Moderately suitable habitat		Highly suitable habitat		Total suitable habitat	
		Area (×10^4 km^2)	Change (%)	Area (×10^4 km^2)	Change (%)	Area (×10^4 km^2)	Change (%)	Area (×10^4 km^2)	Change (%)
Current		10.07		20.11		7.10		37.28	
SSP126	2050s	11.29	12.10	22.21	10.41	7.17	1.00	40.66	9.08
	2070s	10.94	8.64	22.42	11.49	8.96	26.24	42.32	13.53
SSP370	2050s	13.33	32.36	26.09	29.74	6.49	-8.51	45.92	23.16
	2070s	15.94	58.33	28.27	40.54	7.40	4.30	51.61	38.44
SSP585	2050s	11.88	17.97	22.41	11.42	8.10	14.04	42.38	13.69
	2070s	15.07	49.64	25.43	26.46	8.77	23.53	49.27	32.16

Table S2. Centroid coordinates and migration distance of *C. indica*, *C. hystrix*, *S. wallichii* forest under climate change.

Periods-Scenarios	Longitude	Latitude	Migration distance compared with the previous period
Current	100°41'E	27°38'N	
2050s-SSP126	100°11'E	28°31'N	109.07
2050s-SSP370	100°15'E	29°00'N	157.25
2050s-SSP585	99°45'E	28°35'N	139.26
2070s-SSP126	99°38'E	28°36'N	54.66
2070s-SSP370	98°24'E	29°07'N	181.48
2070s-SSP585	98°03'E	29°29'N	193.72

Table S3. The suitable habitat distribution of *C. indica*, *C. hystrix*, *S. wallichii* forest after incorporating dispersal limitations.

Scenarios	Periods	Barriers and unsuitable areas		Occupied suitable areas		Unoccupied suitable areas		Comparison with the suitable habitat area under climate change
		Area (×10^4 km^2)	Ratio (%)	Area (×10^4 km^2)	Ratio (%)	Area (×10^4 km^2)	Ratio (%)	Ratio (%)
SSP126	2050s	935.56	97.37	24.38	2.54	0.90	0.09	-40.05
	2070s	934.72	97.28	25.22	2.62	0.90	0.09	-40.42
SSP370	2050s	932.86	97.09	25.90	2.70	2.09	0.22	-43.60
	2070s	930.50	96.84	27.54	2.87	2.80	0.29	-46.64
SSP585	2050s	934.86	97.30	25.10	2.61	0.89	0.09	-40.79
	2070s	931.61	96.96	26.54	2.76	2.69	0.28	-46.13

Table S4. Centroid coordinates and migration distance of *C. indica*, *C. hystrix*, *S. wallichii* forest with dispersal limitations incorporated.

Periods-Scenarios	Longitude	Latitude	Migration distance compared with the previous period
Current	100°41'E	27°38'N	
2050s-SSP126	101°02'E	26°43'N	107.87
2050s-SSP370	101°18'E	26°44'N	116.27
2050s-SSP585	101°17'E	26°38'N	126.09
2070s-SSP126	101°02'E	26°38'N	8.90
2070s-SSP370	101°12'E	26°46'N	9.53
2070s-SSP585	100°47'E	26°50'N	53.46

Table S5. Comparison of the results of the MaxEnt model parameter optimization.Berlin/Heidelberg, Germany,

Model evaluation	Feature combination	Regularization multiplier	the delta AICc
Default	LQHP	1	1446.441
Optimised	LQHPT	0.5	0

Table S6. AUC and TSS values of the optimised MaxEnt model.

Model	AUC	TSS
Optimised MaxEnt	0.889	0.759

Table S7. 28 initial environmental variables in the application of the MaxEnt model.

Symbol	Variables Name	Unit	Symbol	Variables Name	Unit
bio1	Annual mean temperature	℃	bio15	Precipitation seasonality	unitless
bio2	Mean diurnal range	℃	bio16	Precipitation of wettest quarter	mm
bio3	Isothermality	unitless	bio17	Precipitation of driest quarter	mm
bio4	Temperature seasonality	unitless	bio18	Precipitation of warmest quarter	mm
bio5	Max temperature of the warmest month	℃	bio19	Precipitation of coldest quarter	mm
bio6	Minimum temperature of the coldest month	℃	elev	Elevation	m
bio7	Temperature annual range	℃	slope	Slope	°
bio8	Mean temperature of the wettest quarter	℃	aspect	aspect	°

bio9	Mean temperature of driest quarter	℃	awc	AWC for rootable soil depth	mm
bio10	Mean temperature of the warmest quarter	℃	ph_water	pH in water	-log(H+)
bio11	Mean temperature of the coldest quarter	℃	sand	sand content	% weight
bio12	Annual precipitation	mm	clay	clay content	% weight
bio13	Precipitation of the wettest month	mm	total_n	Total nitrogen content	g/kg
bio14	Precipitation of the driest month	mm	org_carbon	Organic Carbon Content	% weight
